# Supplementary figures and images for: Association between clinical, serological, functional and radiological findings and ventilatory distribution heterogeneity in patients with rheumatoid arthritis
Source: PLoS One. 2023 Oct 20;18(10):e0291659. doi: 10.1371/journal.pone.0291659 (PMC10588833; doi:10.1371/journal.pone.0291659)

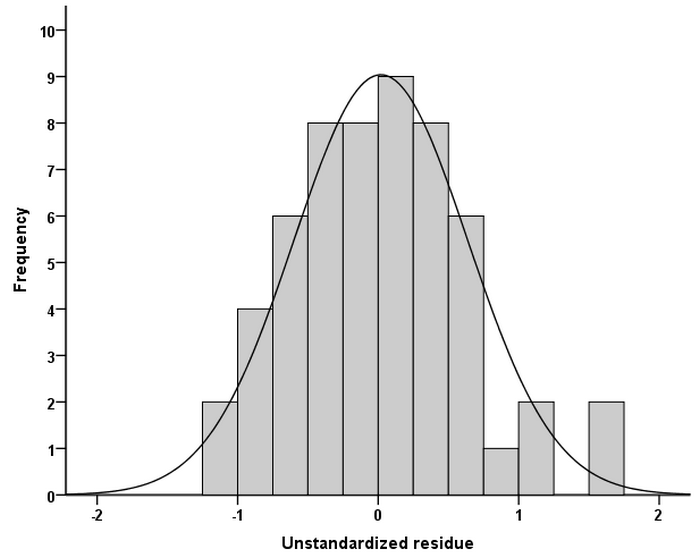

Supplement: S1 Fig — The distribution of unstandardized residuals was approximately normal (p = 0.48). (TIF) [file pone.0291659.s001.tif]

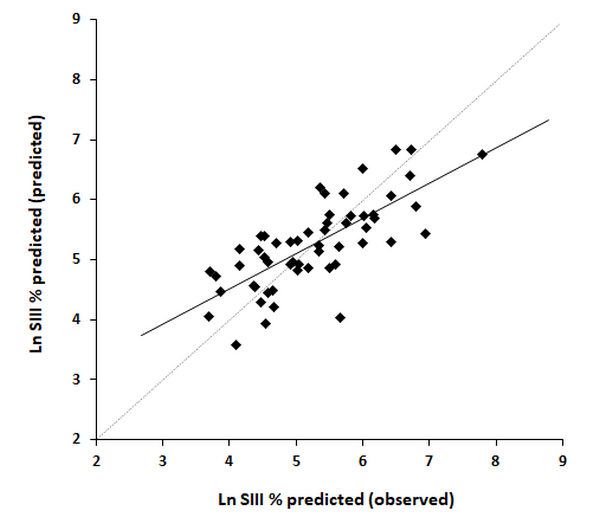

Supplement: S2 Fig — There is a narrow slope between the adjusted regression line and the main diagonal, and a strong correlation between the observed and the respective predicted data (r = 0.74; p<0.0001). (TIF) [file pone.0291659.s002.tif]

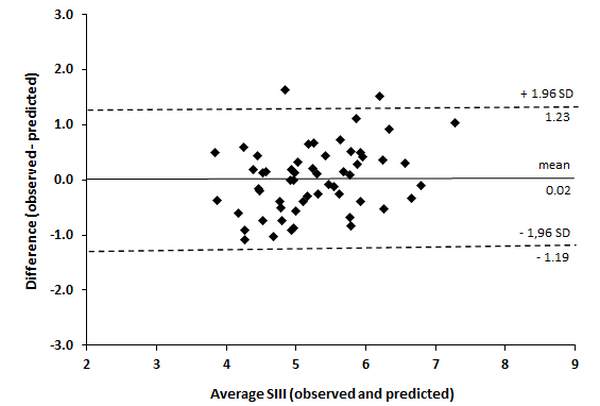

Supplement: S3 Fig — The mean difference was 0.02 with a standard deviation of 0.62, obtaining relatively narrow 95% intervals of agreement (-1.19 for lower and 1.23 for higher). (TIF) [file pone.0291659.s003.tif]
